# HCMV Spread and Cell Tropism are Determined by Distinct Virus Populations

**DOI:** 10.1371/journal.ppat.1001256

**Published:** 2011-01-13

**Authors:** Laura Scrivano, Christian Sinzger, Hans Nitschko, Ulrich H. Koszinowski, Barbara Adler

**Affiliations:** 1 Max von Pettenkofer-Institut für Virologie, Ludwig-Maximilians-Universität München, München, Germany; 2 Institut für Medizinische Virologie, Eberhard Karls Universität Tübingen, Tübingen, Germany; University of Alabama at Birmingham, United States of America

## Abstract

Human cytomegalovirus (HCMV) can infect many different cell types *in vivo*. Two gH/gL complexes are used for entry into cells. gH/gL/pUL(128,130,131A) shows no selectivity for its host cell, whereas formation of a gH/gL/gO complex only restricts the tropism mainly to fibroblasts. Here, we describe that depending on the cell type in which virus replication takes place, virus carrying the gH/gL/pUL(128,130,131A) complex is either released or retained cell-associated. We observed that virus spread in fibroblast cultures was predominantly supernatant-driven, whereas spread in endothelial cell (EC) cultures was predominantly focal. This was due to properties of virus released from fibroblasts and EC. Fibroblasts released virus which could infect both fibroblasts and EC. In contrast, EC released virus which readily infected fibroblasts, but was barely able to infect EC. The EC infection capacities of virus released from fibroblasts or EC correlated with respectively high or low amounts of gH/gL/pUL(128,130,131A) in virus particles. Moreover, we found that focal spread in EC cultures could be attributed to EC-tropic virus tightly associated with EC and not released into the supernatant. Preincubation of fibroblast-derived virus progeny with EC or beads coated with pUL131A-specific antibodies depleted the fraction that could infect EC, and left a fraction that could predominantly infect fibroblasts. These data strongly suggest that HCMV progeny is composed of distinct virus populations. EC specifically retain the EC-tropic population, whereas fibroblasts release EC-tropic and non EC-tropic virus. Our findings offer completely new views on how HCMV spread may be controlled by its host cells.

## Author Summary

gH/gL complexes of herpesviruses are supposed to promote fusion of the viral envelope with cellular membranes. The gH/gL core complex associates with additional proteins which define the tropism for certain cell types by promoting binding to specific receptors. Two alternative gH/gL complexes of human cytomegalovirus (HCMV) define the cell tropism, the entry pathway and the spread of virus. Formation of a gH/gL/gO complex during infection determines release of infectious virus into the supernatant. The gH/gL/pUL(128,130,131A) complex determines the tropism for endothelial cells (EC) and promotes focal spread. Here, we could show that HCMV-infected cells produce EC-tropic and non EC-tropic virus populations. While fibroblasts release both populations into the supernatant, EC predominantly release the non EC-tropic population. Different host cells of HCMV thus may direct the distribution of virus progeny.

## Introduction

Human cytomegalovirus (HCMV) is ubiquitously distributed in the human population. In immunocompetent adults infections are mainly asymptomatic, but in immunocompromised patients like transplant recipients or AIDS patients life threatening infections occur at a high rate. HCMV is also the leading cause of birth defects among congenitally transmitted viral infections. HCMV replicates *in vivo* and *in vitro* in many different host cells including epithelial cells, connective tissue cells, hepatocytes, various leukocyte populations and vascular endothelial cells (reviewed in [1]). The broad host cell range implicates that either an ubiquitous cellular receptor, recognized by one protein or protein complex in the viral envelope, mediates entry, or that HCMV uses elaborate combinations of different viral envelope proteins to employ different cellular receptors. More than 10 glycoproteins have been identified in HCMV particles [2], including the essential glycoproteins gB, gH, gL, gM and gN, which all play a role in the virus entry process [3]–[7]. Although a number of cellular surface proteins have been identified to bind these envelope proteins and play a role in virus particle attachment or promoting intracellular signaling after binding [8]–[13], none of them is currently considered to be a functional entry receptor.

The best candidates for binding to entry receptors are the HCMV gH/gL complexes. The gH/gL complex has been shown to promote fusion of cellular membranes [7] and can either form a gH/gL/gO [14], [15] or a gH/gL/pUL(128,130,131A) complex [16]–[18]. HCMV isolates from patients are consistently able to form both gH/gL complexes [19], [20]. In contrast, many HCMV laboratory strains express only the gH/gL/gO complex, which restricts virus entry to few cell types like fibroblasts and neuronal cells [21], [22]. Leukocytes, dendritic, epithelial and endothelial cells (EC) can only be infected by virus expressing the gH/gL/pUL(128,130,131A) complex [16], [17], [22], [23], which can also promote infection of fibroblasts [24]. Virus strains expressing only gH/gL/gO enter fibroblasts through fusion at the plasma membrane [25]. When fibroblast infection is promoted by gH/gL/pUL(128,130,131A) only, then entry is through pH-sensitive endocytosis [26].

It is currently not clear whether gH/gL/gO complexes exert their function by directly initiating entry [27]. gO has been shown to be incorporated in the virus envelope of the HCMV strain AD169, a laboratory strain which does not express the gH/gL/pUL(128,130,131A) complex [2], [27], but not in the envelope of the clinical isolate TR [27]. Deletion of gO in a virus background, which still allows formation of the gH/gL/pUL(128,130,131A) complex, strongly impairs release of infectious virus particles from infected cells. Virus spread becomes focal and dependent on the gH/gL/pUL(128,130,131A) complex [24], [26], [28].

In contrast to the gH/gL/gO complex, the gH/gL/pUL(128,130,131A) complex has been found to be consistently incorporated into virions [16]–[18], [29]. The exact roles of the individual proteins of the gH/gL/pUL(128,130,131A) complex are not known, but pUL128, pUL130 and pUL131A are all needed to form a functional complex with gH/gL and to have this complex incorporated into virions [16]–[18]. Although the data are controversal, the gH/gL/pUL(128,130,131A) complex very likely promotes entry into endothelial and epithelial cells through an endocytotic pathway [30]–[33]. There is also good evidence for epithelial cells that binding and uptake of virus is promoted through a cell type-specific receptor for the gH/gL/pUL(128,130,131A) complex [34].

Viruses lacking both, gO and pUL(128,130,131A), are not viable, indicating that at least one of the two gH/gL complexes is needed for infection [24]. It is not known whether both gH/gL complexes are incorporated in one particle or whether they are incorporated into distinct particles, and how the usage of the complexes for entry is regulated.

The formation of distinct gH/gL complexes is not restricted to HCMV and has also been described for EBV and HHV-6 [35], [36]. For EBV, a gH/gL/gp42 and a gp42-negative gH/gL complex have been described. The latter binds to integrins α_v_ß_6_ and α_v_ß_8_ and promotes entry into epithelial cells by fusion at the plasma membrane [37]–[39]. The gH/gL/gp42 complex binds to HLA-DR ß and promotes entry into B-cells by an endocytotic route [38]–[40]. During virus production in B-cells, gp42 is intracellularly targeted to HLA-DR ß, where it is vulnerable for degradation. Consequently, B-cells release virus particles, which are low in gH/gL/gp42. This virus is directed towards epithelial cells. Epithelial cells on the other hand do not express HLA-DR ß and produce virus which is high in gH/gL/gp42 and is directed to B-cells [35]. Thus, the EBV host cell tropism is switched by alternate replication in B- or epithelial cells. For HHV-6 a gH/gL/gO and a gH/gL/Q1/Q2 complex have been identified [36], [41], [42]. The latter has a high affinity for the HHV-6 cellular receptor CD46 [41], whereas the gH/gL/gO complex does not bind CD46 [36].

Here, we show that, similar to EBV, also HCMV progenies derived from different cell types differ in their cell tropism. Fibroblast-derived virus progeny could readily infect fibroblasts and EC, whereas EC-derived virus progeny was barely able to infect EC, and this difference in tropism was reflected by a respectively high or low content of the gH/gL/pUL(128,130,131A) complex in virus particles. EC-tropism could be depleted from fibroblast-derived virus progeny, indicating that this progeny is composed of distinct populations of virus particles with different EC infection capacities. Spread patterns in culture and cell disruption experiments indicated that fibroblasts readily released EC-tropic and non EC-tropic virus particles, whereas EC selectively retained the EC-tropic population.

## Results

### HCMV shows different spread patterns in fibroblast and EC cultures

**Figure 1:**
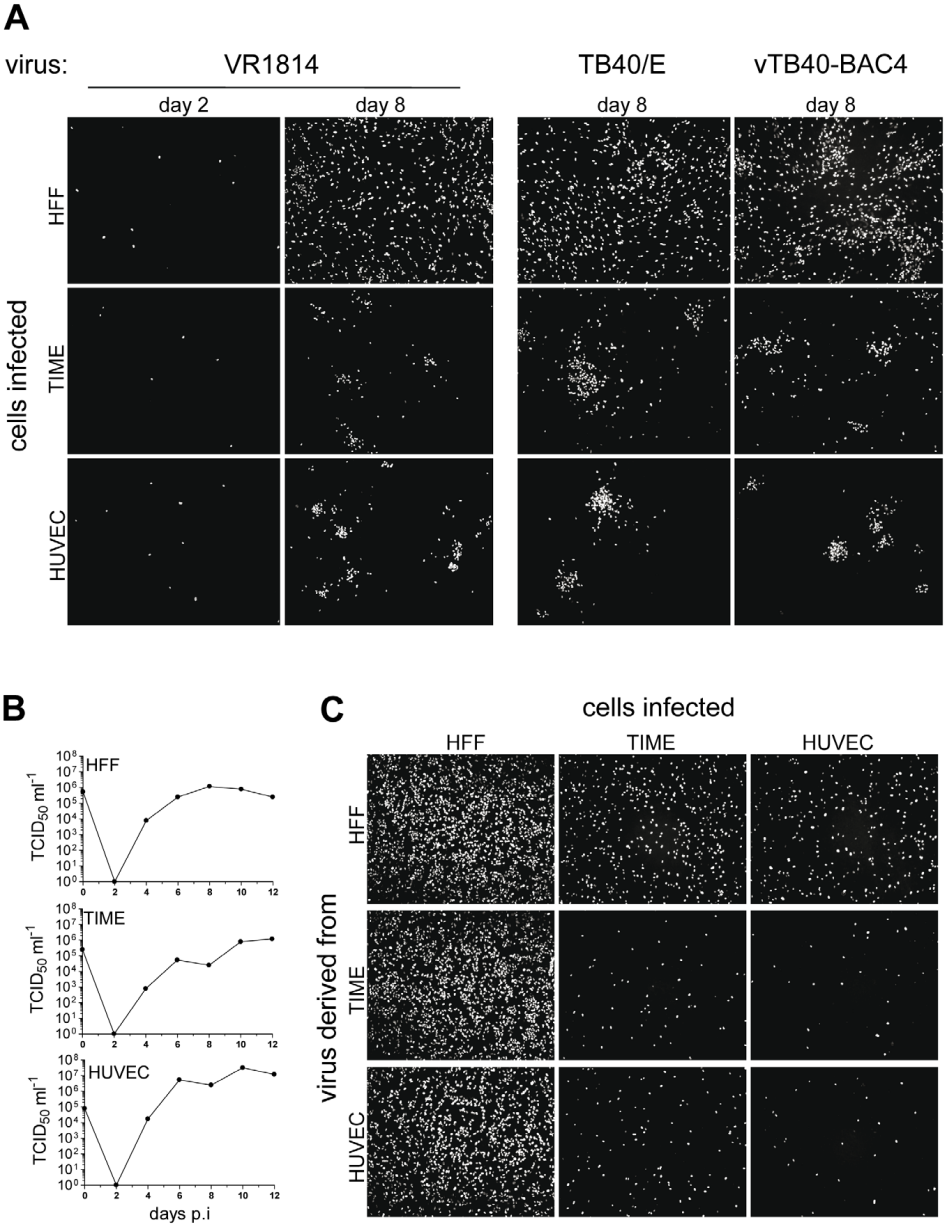
HCMV spread in fibroblast and EC cultures. (A) HFF, TIME cells and HUVEC were infected with VR1814, TB40/E and vTB40-BAC4 as described in Materials and Methods to obtain equal numbers of initially infected cells (m.o.i. on HFF: 0.1). The initial infection (day 2) as well as virus spread (day 8) were monitored by staining for HCMV ie1 protein expression. (B) Growth curves of vTB40-BAC4 on HFF, TIME cells and HUVEC. Cells were infected as described in Materials and Methods to obtain equal numbers of initially infected cells (m.o.i. on HFF: 1). Cell culture supernatants were harvested at the indicated time points post infection and virus titers determined by a TCID_50_ assay performed on HFF. (C) Infection capacities of supernatant-derived vTB40-BAC4. HFF, TIME cells and HUVEC were infected at an m.o.i. of 1 with day 8 supernatants obtained from the growth curves under (B). Infection capacities were monitored by staining for ie1 protein expression 48 hours post infection. Except where indicated, supernatants used for infection were titrated by a TCID_50_ assay on HFF.

When fibroblasts and EC are infected with HCMV *in vitro*, virus homogeneously spreads in fibroblast cultures whereas spread in endothelial cell cultures stays focal [17], [43]. Here, we infected fibroblasts and EC with the HCMV strains VR1814 and TB40/E, two clinical isolates passaged on endothelial cells, and vTB40-BAC4, a virus derived from TB40/E and cloned as a bacterial artificial chromosome (BAC). Infections were performed at a low multiplicity of infection (m.o.i.), and 2 or 8 days after infection cells were stained for HCMV immediate early 1 (ie1) protein expression. Numbers of initially infected HFF or EC were comparable (Fig. 1A, VR1814, day 2 and data not shown). When fibroblasts were infected, HCMV homogeneously spread throughout the culture indicating release of virus from infected cells and infection via free supernatant virus (Fig. 1A, day 8). In contrast, EC infection remained focal indicating virus transmission which delivers virus particles from cell-to-cell, without releasing it. This spread pattern in EC cultures was comparable for all HCMV strains tested and independent of whether a microvascular cell line (TIME) or primary macrovascular endothelial cells (HUVEC) were infected (Fig. 1A, day 8, lower panels). Focal spread in EC cultures could be completely inhibited by neutralizing anti-HCMV antibodies in human serum or anti-pUL131A antibodies (Fig. S1), indicating that virus spread in EC cultures was not due to direct cell-to-cell spread. Spread in fibroblast cultures was restricted from supernatant-driven spread to focal spread by human antiserum and not inhibited at all by anti-pUL131A antibodies (Fig. S1).

To test whether the focal spread could be attributed to differences in release of infectious virus, we performed growth curves of vTB40-BAC4 on HFF, TIME cells and HUVEC, and measured virus release into the supernatants by titration on fibroblasts. HFF and EC equally released high amounts of virus into their supernatants (Fig. 1B). As spread of infection in EC cultures was focal although EC released virus in abundance, focal spread might be due to the inability of EC supernatant virus to infect EC. Indeed, although HFF and EC supernatants comparably infected fibroblasts, the capacities of EC-derived supernatants to infect EC were very low (Fig. 1C). In the experiment shown, spread in HUVEC cultures appeared more cell-associated than in TIME cell cultures, where also single cells in between foci were ie1-positive (Fig. 1A). This correlated with the lower HUVEC infection capacity of EC-derived supernatants when compared to the TIME cell infection capacity (Fig. 1C).

### Quantification of HCMV infection capacities using a TB40-BAC4-derived virus expressing firefly luciferase

Infection capacities on different cell types are often compared by methods, which depend on counting infected cells which are either stained for viral antigen or GFP- expression. These methods reach their technical limits, when the infection capacities strongly differ on the cell types to be tested. It is very difficult to obtain reliable cell counts on the less permissive cell type, without at the same time saturating infection on the more permissive cells. Saturation yet, would lead to an overestimation of the infection capacity on the less permissive cells, when related to the more permissive cells.

To circumvent these problems and to simplify the analysis, we used a luciferase reporter virus to monitor infection. An SV40 promoter-driven luciferase expression cassette was inserted into BAC4-FRT5-9, a TB40-BAC4-derived BACmid lacking the genes UL5 to UL9 and carrying an FRT site at the position of the deleted locus (Fig. S2A). Virus was reconstituted from BAC4-FRT5-9 (vBAC4-FRT5-9) and BAC4-luc (vBAC4-luc). Virus growth of these mutants in HFF and EC was comparable to growth of the parental vTB40-BAC4 (Fig. S2B). Both, vBAC4-FRT5-9 and vBAC4-luc, also showed comparable spread patterns in HFF and EC cultures (Fig. S2C).

**Figure 2:**
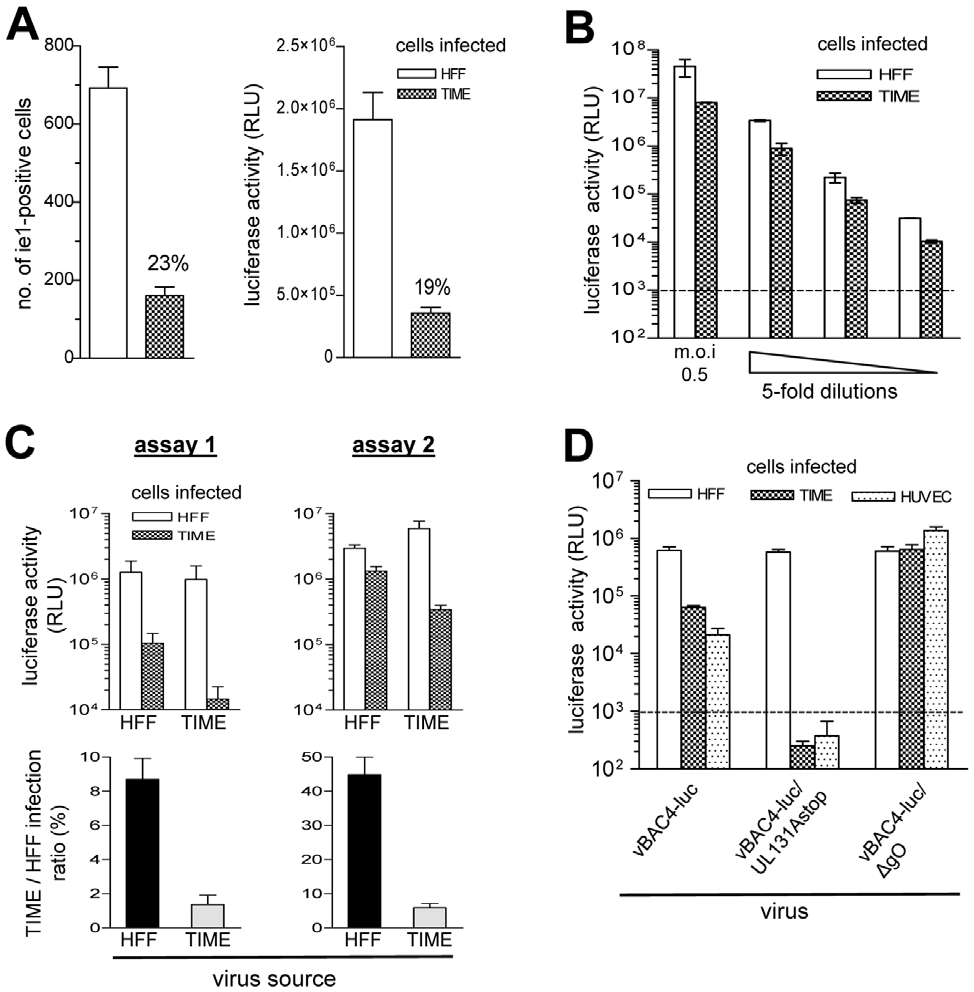
vBAC4-luc as a tool to study infection capacities. (A) HFF and in parallel TIME cells were infected on 96 well plates at an m.o.i. of 0.3 with a cell culture supernatant derived from HFF infected with vBAC4-luc. After infection, PAA was added and 48 hours later cells either stained for HCMV ie1 protein expression or lysed and subjected to a luciferase assay. The experiment was performed in triplicates. For ie1 staining, three independent wells were infected and one microscopic field per well was counted. For the luciferase assay, lysates from three wells were analysed. Shown are means +/− SD of these triplicates. The TIME cell infection capacity was related to the HFF infection capacity which was set to 100%, and the ratio expressed in percent. (B) HFF and TIME cells were infected with serial 5-fold dilutions of a supernatant derived from HFF infected with vBAC4-luc starting at an m.o.i of 0.5 and 48 hours later analysed by a luciferase assay performed in triplicates. The background level of the luciferase assay is indicated by the dotted line. (C) Two independent luciferase assays using different batches of HFF and TIME cells. Three TIME cell and three HFF supernatants from three independent infections with vBAC4-luc were assayed. For the luciferase assay, cells were infected at an m.o.i. of 0.1 and analysed in triplicates 48 hours after infection. Shown are means +/− SD of three supernatants tested in triplicates. (D) HFF, TIME cells and HUVEC were infected at an m.o.i. of 0.02 (centrifugal enhancement) with supernatants from vBAC4-luc, vBAC4-luc/UL131Astop and vBAC4-luc/ΔgO infections of HFF. 48 hours after infection cells were subjected to a luciferase assay. Shown are means +/− SD of luciferase activities determined in triplicates.

We used vBAC4-luc to evaluate EC and fibroblast infection capacities of virus preparations on HFF and TIME cells. The luciferase signals obtained from HFF and TIME cell infections were related to each other and expressed as TIME/HFF infection ratios, and thus, represent relative EC infection capacities. After infection, phosphono acetic acid (PAA) was added to block the viral DNA replication and the further amplification of the luciferase signal. Thus, the luciferase activity evaluates infection of cells in a fashion analogous to staining cells for HCMV ie1 protein expression. Indeed, when infection with one and the same virus preparation was evaluated either by counting ie1-positive cells or by measuring the luciferase activity in cell lysates, both methods always gave comparable results (Fig. 2A and data not shown). The assay proved to be linear over a wide range of m.o.i and highly sensitive (Fig. 2B).

It is a standard observation in the field that one and the same HCMV preparation yields variable results, when repeatedly titrated on different target cell batches. When we tested HFF- and TIME cell-derived supernatants in independent luciferase assays, the results strongly depended on the quality of the cells used and varied with e.g. passage number and time after passage (Fig. 2C). Virus preparations derived from infected HFF and TIME cells (virus source) were tested twice, using different batches of HFF and TIME cells (assay 1 and 2). In assay one, the infection capacities of the supernatants on HFF and TIME cell differed much more than in assay two (Fig. 2C, upper panel), and consequently, the TIME/HFF infection ratios were in the range of 8 and 1.5% in assay one and in the range of 40 and 6% in assay two. (Fig. 2C, lower panel). Yet, when the TIME/HFF infection ratios of the HFF supernatants were divided by the TIME/HFF infection ratios of the TIME cell supernatants, the quotients were comparable in both assays (assay one: 6.9, assay two: 7.5). Therefore, the properties of virus preparations to be compared to each other were always tested in parallel.

EC infection strictly depends on the gH/gL/pUL(128,130,131A) complex [16], [17]. The mutant vBAC4-luc/UL131Astop does not express pUL131A. The gH/gL/pUL(128,130,131A) complex is not formed, and the mutant cannot infect EC. The mutant vBAC4-luc/ΔgO does not express gO and promotes entry into EC and also HFF via the gH/gL/pUL(128,130,131A) complex [24], [26]–[28]. We compared both mutants and the parental vBAC4-luc in the luciferase assay. Confirming the data from Figure 1C, supernatant from a vBAC4-luc infection of HFF showed a lower capacity to infect EC, when compared to the capacity to infect HFF (Fig. 2D). vBAC4-luc/UL131Astop infected HFF, whereas the luciferase signals obtained from infected HUVEC and TIME cells remained below the detection limit. vBAC4-luc/ΔgO equally well infected HFF, TIME cells and HUVEC and thus showed an infection pattern clearly different from the parental vBAC4-luc. Taken together, the luciferase assay proved to be highly sensitive, to allow quantitative measurements over a wide range of m.o.i., and to reflect what is seen, when infection is detected by staining cells for ie1 protein expression.

**Figure 3:**
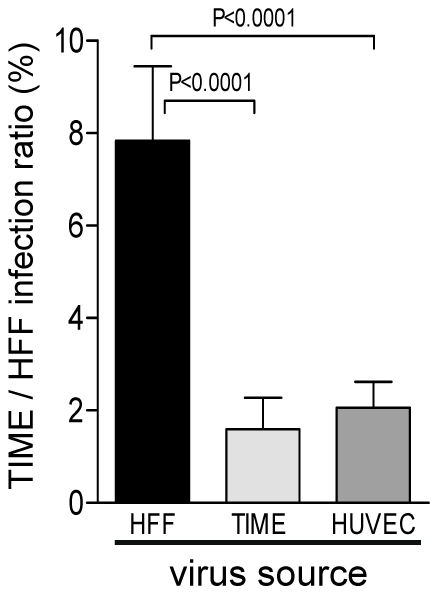
Virus released from EC shows a lower capability to infect EC than virus released from fibroblasts. HFF, TIME cells and HUVEC were infected with vBAC4-luc as described in Materials and Methods to obtain equal numbers of initially infected cells (m.o.i. on HFF: 1). Supernatants from these infections were harvested 6 days after infection and titrated. HFF and TIME cells were then infected with these supernatants at an m.o.i. of 0.1. After infection, PAA was added to the cultures, and 48 h later, cells were lysed and subjected to a luciferase assay. The capacity of supernatant virus to infect EC was expressed as the ratio of TIME cell infection to HFF infection. HFF infection was set to 100%. Shown are means +/−SD of at least five independent experiments. EC-derived supernatants were significantly less capable to infect EC than fibroblast-derived supernatants (Student's t test).

With the luciferase assay described above, we could compare the properties of virus progenies from HFF and EC. Supernatants of infected HFF and EC were harvested 6 days after infection, titrated on HFF, and the viral DNA content determined by real-time PCR. The ratios of infectious virus to viral DNA copy numbers were comparable for HFF- and EC-derived supernatants (data not shown). These supernatants were then used to infect HFF and TIME cells. Forty-eight hours later, infection was monitored by the luciferase assay. Although virus derived from all three cell types showed a comparable infection of HFF (data not shown), EC-derived virus was significantly less capable in infecting EC than fibroblast-derived virus (Fig. 3). On average, the TIME/HFF infection ratios were about fourfold lower for virus released from EC than for virus released from HFF.

### The capacity of supernatant virus to infect EC correlates with UL128 protein content in virions

**Figure 4:**
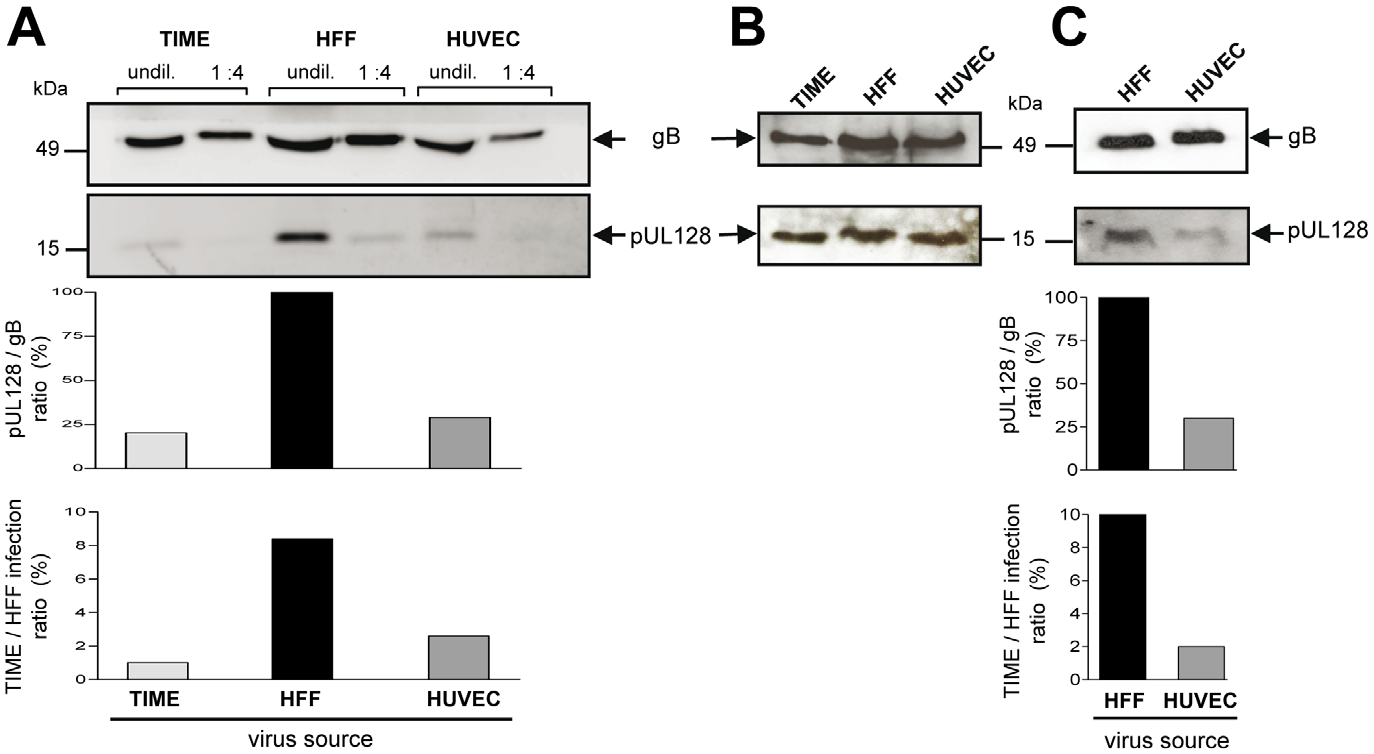
EC infection capacities correlate with pUL128 protein content of virus particles. HFF, TIME cells and HUVEC were infected with vTB40-BAC4 or vBAC4-luc for the gradient purified virus as described in Materials and Methods to obtain equal numbers of initially infected cells (m.o.i. on HFF: 1). Supernatants were harvested, when cells showed about 80% CPE. (A) Supernatant-derived particles were concentrated by ultracentrifugation and pUL128 and gB protein levels detected by Western blot analysis of undiluted or 1∶4 diluted lysates. The pUL128 band intensities of undiluted samples are expressed as ratios of pUL128 band intensities to gB band intensities. This ratio was set to 100% for the HFF-derived particles and the ratios for HUVEC and TIME cell-derived particles expressed relative to the HFF value (middle panel). The virus preparations were additionally tested for their TIME/HFF infection ratios by staining infected HFF and TIME cells for ie1 protein expression 48 h after infection (lower panel). (B) Cellular expression levels of gB and pUL128 were monitored by Western blot analysis of total cell lysates of infected cells. (C) Analysis of gradient-purified virus derived from HFF and HUVEC infections. Cells were infected as described above. Virus was purified from the supernatants on glycerol/tartrate gradients as described in Materials and Methods and virus preparations analysed as described under (A).

Incorporation of gH/gL/pUL(128,130,131A) glycoprotein complexes into virions [16], [17] is a prerequisite to infect endothelial cells. As virus released from EC was less capable in infecting EC than virus released from fibroblasts, we asked whether this difference can be associated with the abundance of gH/gL/pUL(128,130,131A) complexes incorporated into virions. We determined the gB and gH levels, and, representative for the presence of the gH/gL/pUL(128,130,131A) complex, the pUL128 content in EC- and HFF-derived virions. Virus particles were pelleted from EC- or HFF-derived supernatants, lysed, and their gB, gH and pUL128 protein content determined by Western blot analysis. The amounts of gB and gH in virus pellets from HFF and EC supernatants always showed a constant relation (data not shown). Yet, HFF-derived virus particles contained more pUL128 protein than EC-derived virus particles (Fig. 4A). This could be quantitatively analysed by measuring the gB band intensities of the lysates, which reflect the particle amounts loaded, and then, relating the pUL128 band intensities to the respective gB bands (Fig. 4A, middle panel). Remarkably, the pUL128/gB ratios mirror the TIME/HFF infection ratios (Fig. 4B, lower panel). Thus, a low EC infection capacity correlated with a low level of gH/gL/pUL(128,130,131A) complexes in virions. Interestingly, total cell lysates of the respective infected cells showed that EC and HFF expressed comparable amounts of pUL128 (Fig. 4B). This indicated that the differences in EC infection capacities observed are created at a late stage during maturation or release of virus progeny. To exclude that the observed differences between EC- and HFF-derived supernatants are due to non-infectious particles or contaminations with cell membrane components, gradient-purified virus from infections of HFF and HUVEC were analysed in the Western blot as described above (Fig. 4C). The pUL128/gB ratios again mirrored the TIME/HFF infection ratios of the supernatants, the virus was purified from.

### Endothelial cells produce, but not readily release EC-tropic HCMV

**Figure 5:**
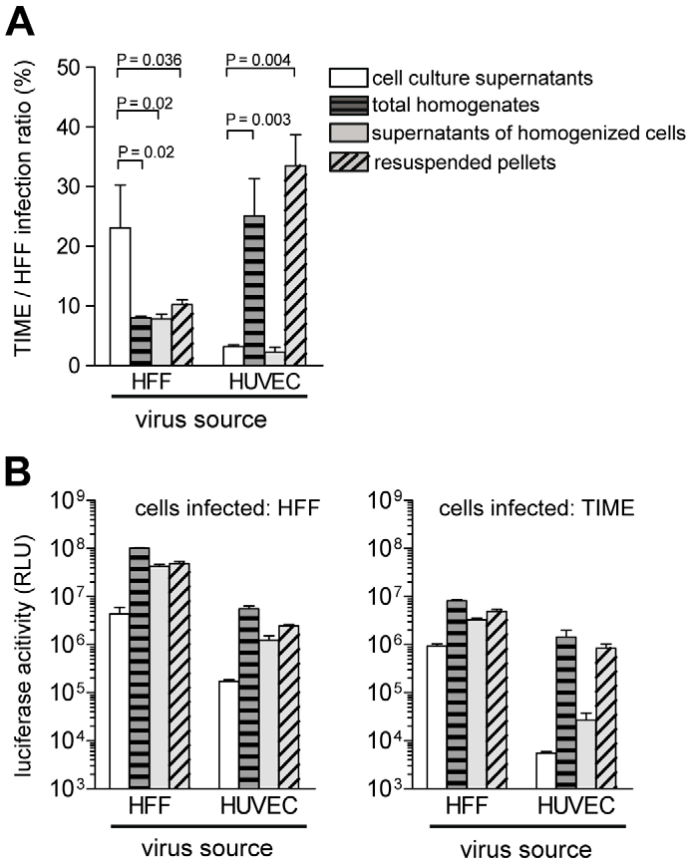
Endothelial cells retain EC-tropic virus particles. HFF were infected with vBAC4-luc as described in Materials and Methods to obtain equal numbers of initially infected cells (m.o.i. on HFF: 0.2). Six days after infection supernatants were harvested, cells homogenized as described in Materials and Methods and different fractions of the homogenates tested on HFF and TIME cells for their infection capacities by luciferase assay. (A) shows the TIME/HFF infection ratios and (B) the absolute luciferase activities of the different cell preparations on HFF and TIME cells. Shown are means +/− SD of three independent experiments assayed in triplicates. For HFF all homogenate preparations were significantly less EC-tropic than the cell culture supernatants. For HUVEC the total homogenates and the resuspended pellets were significantly more EC-tropic than the cell culture supernatants (Student's t test).

Whereas the virus released by EC is low in gH/gL/pUL(128,130,13A) complexes, focal spread in EC cultures was highly efficient. Like EC infection by supernatant virus, it can be blocked by anti-pUL128, anti-pUL130 and anti-pUL131A antibodies [16], [17], [44], [45]. This indicates that pUL(128,130,131A) are accessible to antibodies and promote infection of neighboring cells. We tested different cellular preparations for the presence of cell-associated EC-tropic virus. HUVEC and as a control HFF were infected with vBAC4-luc, and 6 days after infection supernatants were harvested. Cells were washed to remove loosely bound virus and then homogenized using cell douncers. Aliquots of the total homogenates, containing the disrupted cells and virus freed by cell disruption, were saved. Homogenates were then cleared by centrifugation at 3,500×g to separate supernatants containing virus, which can be released by physical disruption. The pellets of cell debris, containing virus which is not released from cells, were also resuspended. These four preparations were then tested on HFF and TIME cells by the luciferase assay. Virus supernatants from HFF and HUVEC showed a high and a low EC infection capacity, respectively (Fig. 5A). All three homogenate preparations from HFF showed a reduced EC infection capacity, when compared to HFF supernatant virus. Notably, the two HUVEC preparations, which contained cell debris showed an about tenfold higher EC infection capacity than the HUVEC supernatants (Fig. 5A). Thus, the progeny able to infect EC is released by HFF, but remained tightly associated with cellular structures in the case of EC. The differences observed are not due to different quantities of virus in the different preparations, because all HFF- and HUVEC-derived preparations showed high luciferase values, when tested on HFF (Fig. 5B). The tenfold differences between HUVEC-derived preparations, containing broken cells, and those without cells are due to high and low luciferase values on TIME cells, respectively (Fig. 5B). Highly EC-tropic virus could neither be released from EC by sonication nor by several rounds of freezing and thawing (data not shown). Taken together, the data show that HFF readily release, whereas HUVEC tightly retain EC-tropic virus.

### HFF release virus progeny, which is not homogeneous

**Figure 6:**
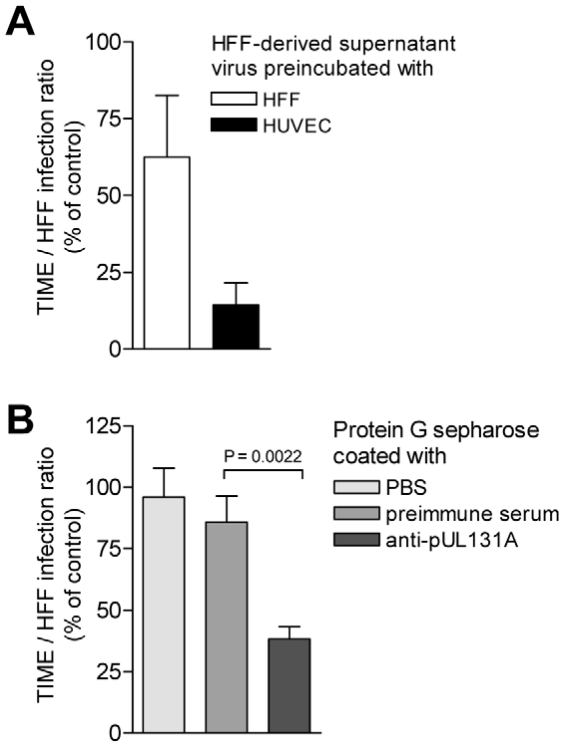
HFF virus progeny can be depleted of EC-tropic virus by coincubation with endothelial cells and beads coated with anti-pUL131A antibodies. (A) 2×10^6^ HFF or HUVEC were incubated (90 min, 37°C) with 130,000 TCID_50_ vBAC4-luc diluted in 30 µl DMEM (5% FCS). Then 500 µl of DMEM (5% FCS) was added, the cells pelleted by centrifugation at 300×g for 5 min and the supernatants cleared at 3,500×g for 15 min. As a control 130,000 TCID_50_ vBAC4-luc diluted in 30 µl DMEM (5% FCS) were mock-incubated and mock-treated as described above. HFF and TIME cells were infected with the supernatants from the coincubations and the controls and subjected to a luciferase assay. TIME/HFF infection ratios of the supernatants after coincubation were expressed as % of the TIME/HFF infection ratios of the control. Shown are the means of TIME/HFF infection ratios +/− SD determined in six independent experiments each assayed in triplicates. (B) 30 µl of a 50% protein G sepharose preparation (GE Healthcare, Germany) were coincubated overnight with 300 µl rabbit anti-pUL131A antiserum (1∶10 diluted in PBS) or as controls preimmune serum (1∶10 diluted in PBS) or PBS. Then, beads were washed 3 times with PBS and coincubated for 2 hours at RT with 130,000 TCID_50_ vBAC4-luc diluted in 500 µl DMEM without serum. After coincubation beads were pelleted and 5% FCS added to the supernatants. Then, HFF and TIME cells were infected with these supernatants and subjected to a luciferase assay. Shown are the means of TIME/HFF infection ratios +/− SD determined in triplicates. The TIME/HFF infection ratios of virus coincubated with anti-UL131A antibody coated beads was significantly different from the values obtained from virus coincubated with preimmune serum-coated beads.

Virus released from EC is only poorly tropic for EC, whereas virus associated with EC shows a much higher tropism for EC. Virus released from HFF is highly EC-tropic and virus found in the particulate fraction of disrupted HFF rather shows a lower tropism for EC. One explanation would be that HCMV progeny is heterogeneous and consists of distinct virus populations with regard to their EC-tropism. EC show a propensity to retain EC-tropic virus and release non EC-tropic virus, whereas HFF readily release both, EC-tropic and non EC-tropic virus. The hypothesis, that HFF progeny is a mixture of EC-tropic and non EC-tropic virus is testable by separation of EC-tropic and non EC-tropic virus. As HUVEC strongly retain EC-tropic virus, they might serve to specifically bind EC-tropic virus and deplete HFF virus progeny of its EC-tropic fraction. We preincubated HFF-derived supernatant virus with HUVEC or with HFF, pelleted the cells, and analysed the HFF and TIME cell infectivity of virus remaining in the supernatants (Fig. 6A). Preincubation with HUVEC removed about 30 to 90% and preincubation with HFF about 99% of the infectious virus from supernatants, when tested on HFF (data not shown). The TIME/HFF infection ratios of the non-bound virus in supernatants preincubated with HUVEC was drastically and significantly reduced to the level observed in supernatants of HCMV-infected HUVEC (Fig. 6A). Preincubation with HFF in contrast, although it removed the bulk of infectivity, only weakly reduced the EC infection propensity of the non-bound virus (Fig. 6A). Thus, HUVEC, which retained EC-tropic virus in infection, were a good matrix for binding EC-tropic virus, whereas HFF, which readily released EC-tropic virus into the supernatant, were a weak matrix for EC-tropic virus. The depletion of EC-tropism strongly suggested that HFF virus progeny was heterogeneous and composed of distinct virus populations, which could be sorted. To find out whether the depletion of EC-tropism is based on removing virus particles expressing the gH/gL/pUL(128,130,131A) complex, we coincubated HFF-derived virus progeny with protein G sepharose beads to which we had bound anti-pUL131A antibodies. Beads coated with antibodies specific for pUL131A, but not uncoated beads or beads coated with preimmune serum, depleted about 70% of the EC-tropism (Fig. 6B). This strongly implied that depletion of EC-tropism is through retaining virions, expressing the gH/gL/pUL(128,130,131A) complex.

## Discussion

The use of different receptor binding proteins to mediate entry into different cell types, and the use of different entry pathways even into one cell type is a common feature of herpesvirus entry. Herpesviruses have additionally developed strategies, which may route infection *in vivo*. For EBV, the group of L. Hutt-Fletcher has pioneered the paradigm that epithelial cells produce a virus progeny high in gH/gL/gp42 complexes, which promotes B-cell infection. B-cells in turn, produce virus progeny low in gH/gL/gp42 complexes which efficiently infect epithelial cells, but not B-cells. Although not absolute, this relative switch of cell tropism after alternate replication in epithelial and B-cells directs infection from one cell type to the other.

Here, we propose that also different producer cells of HCMV may direct the infection. gH/gL/gO complex formation is needed for release of infectious virus from any infected cell type tested so far [24], [28]. Incorporation of gH/gL/pUL(128,130,131A) complexes into virions is essential for infection of e.g. endothelial, epithelial, and dendritic cells, and for leukocytes [16], [17], [22], [23]. If gO is missing, then the infection spreads predominantly focal and depends on the gH/gL/pUL(128,130,131A) complex, even in cell types, which usually do not depend on this complex for infection [24], [26].

Our initial observation was that virus spread in fibroblast cultures differed from virus spread in EC cultures [17]. Spread in fibroblast cultures appeared supernatant- driven, whereas spread in EC cultures was focal. A strictly cell-associated virus spread in EC cultures had also been observed by the group of G. Gerna, who reported that propagation in HUVEC strictly depended on passage of cells and could not be achieved by supernatant virus [43]. We offer an explanation for the focal spread in EC cultures by showing that EC predominantly release virus, which is not EC-tropic, but at the same time tightly retain EC-tropic virus which may then be transferred to neighboring cells only. Virus transfer was accessible to neutralizing antibodies and dependent on the gH/gL/pUL(128,130,131A) complex, as we could show in Figure S1. Focal spread was completely blocked by a neutralizing human antiserum and by anti-pUL131A antibodies. Interestingly, when infected HFF cultures were treated in a similar way, a neutralizing human antiserum blocked infection by free virus, but left a focal spread of virus, indicating that for HFF a direct cell-to-cell spread mechanism may be possible [46]. Anti-pUL131A antibodies could not at all inhibit spread in HFF cultures. These data confirmed earlier studies by our group, which showed that only gH/gL/pUL(128,130,131A) dependent virus spread, like spread in EC cultures, or spread of a delta gO mutant in fibroblast cultures could be inhibited by anti-pUL131A antibodies [17], [26].

Similar to the EBV model, supernatants from infected HFF showed a higher capacity to infect EC than EC-derived supernatants, and we could show that the biochemical basis for that is a respectively high and low content of gH/gL/pUL(128,130,131A) complex in virions.

The question, which arose then, was, what causes the observed difference in gH/gL/pUL(128,130,131A) content. For EBV it has been described that in infected B-cells HLA-DR ß binds the gp42 protein of the gH/gL/gp42 complex, which promotes B-cell infection, holds it back intracellularly, and thus, makes it vulnerable for degradation. As a consequence, B-cells release mainly virions containing a two-part gH/gL complex, which cannot infect B-cells. Epithelial cells, which do not express HLA-DR ß, do not retain gp42 and thus, release virus, which contains more of the three-part gH/gL/gp42 complex. The mechanisms, by which the differences in the released populations of virions in HCMV are achieved, appear to be different. For HCMV, we found that EC and fibroblasts produce heterogeneous virus progenies. EC release a virus progeny, which is not EC-tropic, and retain a progeny, which is highly EC-tropic. HFF release an EC-tropic progeny. which can be depleted of its EC-tropism by using HUVEC or protein G sepharose beads coated with antibodies directed against pUL131A. This strongly suggested that HFF progeny is composed of distinct EC-tropic and non EC-tropic virus populations, and that the EC-tropic population most likely is a population with a high gH/gL/pUL(128,130,131A) content. If HFF-derived virus progeny was homogeneous, a specific depletion only of EC-tropic virus would not be possible. Interestingly, HUVEC, which retain EC-tropic virus in infection experiments, were a good matrix to retain EC-tropic virus in the test tube, whereas HFF, which readily release EC-tropic virus in infections, were a bad matrix.

**Figure 7:**
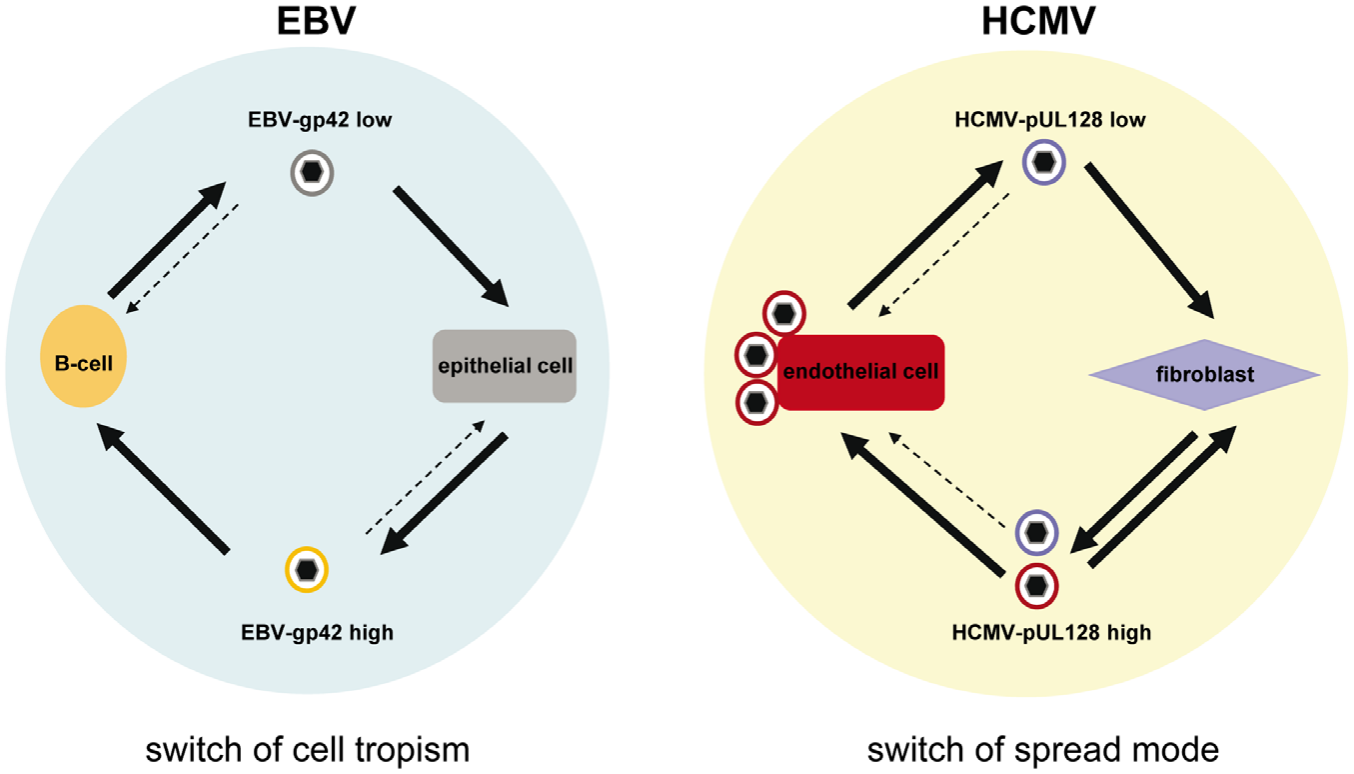
HCMV and EBV models for virus spread in cell culture. The EBV model proposes a switch of cell tropism through depletion of gp42 from B-cell derived virus progeny resulting in a gp42_low_ progeny versus a gp42_high_ progeny released from epithelial cells. The HCMV model proposes the production of a heterogeneous virus progeny from fibroblasts and EC. Fibroblasts release EC-tropic and non-EC tropic viruses which results in a pUL128_high_ progeny, whereas EC release a non EC-tropic pUL128_low_ progeny and retain the EC-tropic virus. This results in a switch of the spread mode. The tropism of virus progenies is indicated by color codes.

Thus, we propose that the difference in cell tropism of virus released from EC and fibroblasts is the result of a sorting process. EC strongly and specifically retain EC-tropic virus through the gH/gL/pUL(128,130,131A) complex. HFF release EC-tropic and non EC-tropic virus. Thus, for HCMV, not protein components of gH/gL complexes are retained in a cell-type specific manner, but rather mature virions carrying the gH/gL/pUL(128,130,131A) complex in their envelopes. Figure 7 depicts the EBV and HCMV models for virus spread side by side.

Future experiments will have to show where and how EC-tropic virus is held back. It has recently been shown that overexpression of gH/gL/pUL(128,130,131A) in epithelial cells interferes with HCMV infection. It has been postulated that this reflects binding of the gH/gL/pUL(128,130,131A) complex to the respective entry receptor [34]. This was not observed for fibroblasts and thus, an HCMV entry receptor binding to gH/gL/pUL(128,130,131A) and expressed on EC would be a good candidate also for retaining EC-tropic virus by infected EC. How virus is then transferred to neighboring cells, will also have to be investigated in the future. An attractive model would be a mechanism as described for MHV-68, for which it has been shown that virus particles attached to and moving on plasma membrane fronds are directly transferred to neighboring cells [47].

Assuming that gH/gL/pUL(128,130,131A) complexes are incorporated into virus progeny at random, EC-tropism of a virus particle might be defined by a threshold level of gH/gL/pUL(128,130,131A) complexes. Accordingly, high levels of gH/gL/pUL(128,130,131A) complexes in turn could also block release from EC. Thus, the levels of gH/gL/pUL(128,130,131A) complexes could define whether a particle is EC-tropic or not, whether it is retained by EC during infection, and whether it can be depleted from supernatants by EC-preincubation. This could also explain, why progeny of a ΔgO virus, which expresses only the gH/gL/pUL(128,130,131A) complex, readily spreads cell-associated and can barely be released from EC [24], [28]. ΔgO virus progeny can equally well infect EC and HFF (Fig 2D). Wildtype TB40-BAC4 virus progeny, in contrast, shows a higher propensity to infect HFF (Fig. 2D), which could be explained by being a mixture of EC-tropic and non EC-tropic particles.

For EBV, it has been observed that virus bound to the surface of resting B cells is 10^3^-10^4^ times more infectious for epithelial cells than cell-free virus [48]. For HCMV it has not yet been tested whether surface-bound virus could promote a switch of cell tropism.

We restricted our experiments to endothelial cells and fibroblasts. Macrophages, dendritic cells, and epithelial cells also strictly depend on the gH/gL/pUL(128,130,131A) complex for their infection. Whether their infection also follows the pattern of the EC infection shown here, will have to be investigated in the future. Recently, it has been published by Wang et al. [30] that HCMV progenies derived from epithelial cells and fibroblast also differ. They reported that both cell types release progenies which can readily infect epithelial cells and fibroblasts, but differ with respect to the pathway they use to enter epithelial cells. They found a twofold higher gH/gL/pUL(128,130,131A) content in epithelial cell-derived particles, which they considered as marginal. As they used an AD169 mutant, in which UL131A had been repaired, it will have to be clarified, whether their findings reflect that epitheliotropic virus produced in epithelial cells is, in contrast to our findings in EC, not retained, or whether the observed differences are due to differences of the HCMV strains used. It has recently been shown that AD169 incorporates gO into virions, whereas HCMV strain TR does not [27]. This suggests that strain-specific differences may indeed affect gH/gL-dependent processes.

Whether our observations made in cell culture, reflect features valid for all HCMV strains, and what role a switch in tropism and spread patterns may play *in vivo*, will be the subject of future research. It will be of particular interest to find out whether the relative propensity of different cell types to release virus plays a crucial role in establishment of infection and transfer of virus to new hosts or the fetus. For HCMV, it has been shown that primary isolation of EC-tropic virus depends on infected cells as a source of virus, whereas fibroblast infection can also be achieved with cell-free virus sources like throat washes and amniotic fluid [43]. This might already be an indication that cells lining the compartments, where these fluids are produced, do not release EC-tropic virus.

## Materials and Methods

### Cells and viruses

Primary human foreskin fibroblasts (HFF) (PromoCell, Germany) were used from passage 12 to 22 and maintained in Dulbecco's modified Eagle's medium (DMEM) supplemented with 10% fetal calf serum, 2 mM L-glutamine, 100 units/ml penicillin and 100 µg/ml streptomycin. Primary human umbilical vein endothelial cells (HUVEC) (LONZA, USA) were used from passage 1 to 6. HUVEC and TIME (telomerase-immortalized human microvascular endothelial) cells [49] were maintained in an EGM-2 MV BulletKit medium system (LONZA, USA).

The HCMV strains used were VR1814 [19], TB40/E [50] and TB40/E cloned as a BAC (TB40-BAC4) [51].

### BAC mutagenesis and construction of recombinant HCMV

The HCMV strain TB40/E cloned as a bacterial artificial chromosome (BAC) (TB40-BAC4) [51] was used for HCMV BAC mutagenesis. A 48 bp FRT site was inserted into the TB40-BAC4, thereby disrupting the open reading frames (ORFs) UL5, UL6, UL7, UL8 and UL9. Briefly, a linear PCR fragment containing a kanamycin-resistance gene flanked by two 48 bp FRT sites and sequences homologous to the HCMV UL5 and UL9 coding regions was generated using the primers UL5pcp15for (5′-ATGTTTCTAGGCTACTCTGACTGTGTAGATCCCGGCTTTGCTGTATATCGTGTATCTAGACGGGGGTGTCCAGGGTTTTCCC-3′) and UL9pcp15rev (5′-ATTGTTGTAACGATAACTAAGGGTATGATCCACATTGTATGTGGGGTGGCAGTATCGTGTCTTCCGGCTCGTATGTTGTGTGG-3) and pCP15 as template [52]. The PCR product was inserted into TB40-BAC4 by homologous recombination in *E. coli*, thereby deleting 3,066 kb. The kanamycin-resistance gene was subsequently excised by *FLP*-mediated site-directed recombination [53], and the resulting BAC mutant called BAC4-FRT5-9.

To generate a luciferase reporter HCMV, the SV40-driven firefly luciferase expression cassette was excised from pGL3-promoter (Promega) with *Sal I* and *Bgl II*, filled in by Klenow polymerase and inserted into the pOriR6K-zeo plasmid linearized by *EcoR V*. The resulting plasmid pO6-Luc was inserted into BAC4-FRT5-9 via *FLP*-mediated FRT recombination mutagenesis using the temperature-sensitive expression plasmid pCP20 [54]. The resulting BAC mutant was called BAC4-Luc.

The BAC mutants BAC4-Luc/ΔgO and BAC4-Luc/UL131Astop were cloned into the BAC4-Luc background as described previously [24], [26].

Deletions and insertions were controlled by restriction pattern analysis and subsequent sequencing.

### Reconstitution of virus from recombinant BACmids

BACmids were reconstituted to virus by transfection of BAC DNA into HFF using FugeneHD transfection reagent (Roche Diagnostics) according to the manufacturer's instructions. Transfected cells were propagated until viral plaques appeared and the supernatants from these cultures used for further propagation of virus.

### Preparation of virus stocks, concentration of virus particles, virus titration and infections

Virus stocks were prepared from supernatants of infected HFF, HUVEC or TIME cells. Supernatants were cleared of cellular debris by centrifugation for 15 min at 3,500×g and stored at −80°C.

For Western Blot analysis of HCMV particles, virus was concentrated from cell culture supernatants. Briefly, 200 ml supernatant from infected cells showing about 90% CPE was cleared of cellular debris by centrifugation at 3,500×g for 15 min. Then, virus was pelleted from cleared supernatant by ultracentrifugation at 80,000×g for 70 min. Virus pellets were resuspended in 1.5 ml 0.04 mol/l sodium phosphate pH 7.4.

Virus titers of cleared supernatants were determined by a TCID_50_ assay performed on 96 well plates on HFF.

To infect cells, medium was removed from 90% confluent cell monolayers and replaced by virus diluted in DMEM containing 5% FCS. For some experiments, virus infection was enhanced by a centrifugation step (30 min, 860×g at room temperature), followed by incubation at 37°C for 90 min. To compare infectivity of virus derived from fibroblasts and endothelial cells, subsequent infections were performed in DMEM 5% FCS/EGM-2 mixed at a ratio of 1∶1, to exclude medium effects. During infections, medium was exchanged every second day in a way that supernatants harvested contained virus released during the preceding 48 hours.

As HCMV in general more readily infects fibroblasts than EC, in all experiments, where infections of EC and fibroblasts (spread patterns and growth curves) were compared, the infections were adapted in a way that EC were infected with more virus than fibroblasts to achieve comparable numbers of ie1-positive cells after 48 hours.

### Gradient purification of virions

For gradient purification of virions, supernatants from infected cell cultures showing approximately 100% late-stage CPE were cleared of cell debris by centrifugation for 10 min at 2,800×g. Supernatants were then ultracentrifuged for 70 min at 80,000×g. Pellets containing virions were resuspended in 1 ml PBS and transferred onto a preformed, linear glycerol/tartrate gradient (15–35% sodium tartrate and 30–40% glycerol in 0.04 mol/l sodium phosphate pH 7.4), which was ultracentrifuged for 45 min at 80,000×g. The virion-containing band was harvested with a syringe and the virions were washed and pelleted by an additional ultracentrifugation for 70 min at 80,000×g. The pellet was resuspended in 0.04 mol/l sodium phosphate.

### Indirect immunofluorescence

HCMV-infected cells were fixed in 50% acetone/50% methanol, stained using a mouse anti-ie1 antibody (anti-ie1; Perkin Elmer) and detected with a Cy3-coupled goat anti-mouse antibody (Dianova). For counterstaining of cell nuclei, cells were incubated in PBS containing 5 µg/ml Hoechst 333258 (Invitrogen) for 1 min.

### Luciferase assay

HFF and TIME cells were grown in 96 well plates (20,000 cells/well) and infected in triplicates at an m.o.i. between 0.02 and 0.5 for 90 min. Inoculi were then replaced by medium supplemented with 300 µg/ml phosphono acetic acid (PAA). 48 h after infection cells were lysed in 50 µl lysis buffer (25 mM Tris/H_3_PO_4_, 2 mM CDTA, 2 mM DTT, 10% glycerol, 5% Triton-X 100) and luciferase activity was determined for 20 µl of lysate with a luciferase assay system (Promega) according to the manufacturer's instructions.

### Western blot analysis

Virus particles or infected cells were lysed in 6× sample buffer (300 mM Tris-HCl (pH 6.8), 10% SDS, 30% glycerol, 5% ß-mercaptoethanol, 0.01% (w/v) bromphenolblue, 0.01% (w/v) phenolred), separated on 15% polyacrylamide gels and transferred onto nitrocellulose (Amersham Biosciences). Membranes were blocked with 5% low-fat milk in TBS and stained for gB or pUL128 using mouse anti-gB antibody (2F12; Abcam) or mouse anti-pUL128 antibody (4B10, kindly provided by T. Shenk, University of Princeton, USA), respectively. The specific protein bands were detected by using an peroxidase-coupled anti-mouse antibody (Dianova) and the SuperSignal West Dura Extended Duration Kit (Perbio).

The intensities of protein bands were quantified using the Fujifilm Intelligent Light Box LAS-300 and the Image reader LAS-300. Non-saturated light signals were analysed to determine the protein amounts using ImageQuant 5.0 software. The pUL128 levels were related to gB levels of the respective samples.

### Homogenization of cells

Cells were infected in 6 cm dishes, and 6 days after infection the supernatants (4 ml) harvested and cleared of cellular debris (3,500×g, 15 min). Cell monolayers were washed with cold PBS, scraped and cells dounced in 4 ml DMEM medium supplemented with 5% FCS using tight fit hand homogenizers (Sartorius-Stedium). 3.5 ml of the total homogenized cells were pelleted (3,500×g, 15 min), the supernatant removed (supernatant of homogenized cells) and the pellets resuspended in fresh 3.5 ml DMEM medium supplemented with 5% FCS.

### Accession numbers

GeneBank/EMBL/DDBJ accession number for TB40-BAC4 is EF999921.

## Supporting Information

Figure S1 Neutralizing antibodies block focal spread of HCMV in EC cultures. HFF and HUVEC were infected with vBAC4-luc as described in Materials and Methods to obtain equal numbers of initially infected cells (m.o.i. on HFF: 0.1). After infection, cells were washed three times with medium and then fresh medium (no serum) or serum diluted in medium was added (1∶25 dilutions of the HCMV-negative and -positive sera and a 1∶10 dilution of the anti-pUL131A rabbit antiserum [17]). Cells were incubated in the presence of the antisera for 8 days. Initial infection (day 2) as well as virus spread (day 8) were monitored by staining for HCMV ie1 protein expression. The HCMV-positive antiserum showed a complete and the HCMV-negative serum no neutralization of HFF and EC infections when tested with free virus (data not shown).
(0.56 MB TIF)

Figure S2 Growth properties of vBAC4-FRT5-9 and vBAC4-luc. (A) Schematic presentation of TB40-BAC4 derived mutants BAC4-FRT5-9 and BAC4-luc. The UL and US regions, the positions of internal and terminal repeats (dark grey), the open reading frames UL5 and UL9, the BAC cassette (light grey), the position of the FRT-site and the insertion of a luciferase expression cassette are indicated. (B) Growth curves of vTB40-BAC4, vBAC4-FRT5-9 and vBAC4-luc on HFF, TIME cells and HUVEC. Cells were infected as described in Materials and Methods to obtain equal numbers of initially infected cells (m.o.i. on HFF: 1). Cell culture supernatants were harvested at the indicated time points post infection and virus titers determined by a TCID50 assay performed on HFF. (C) Spread of vBAC4-FRT5-9 and vBAC4-luc in HFF and endothelial cell cultures. HFF were infected with vBAC4-FRT5-9 and vBAC4-luc at an m.o.i. of 0.1. For TIME cells and HUVEC the m.o.i. were adapted. The initial infections (day 2) as well as virus spread (day 8) were monitored by staining for HCMV ie1 protein expression.
(0.63 MB TIF)
