# Supplementary figures and images for: HCMV Spread and Cell Tropism are Determined by Distinct Virus Populations
Source: PLoS Pathog. 2011 Jan 13;7(1):e1001256. doi: 10.1371/journal.ppat.1001256 (PMC3020925; doi:10.1371/journal.ppat.1001256)

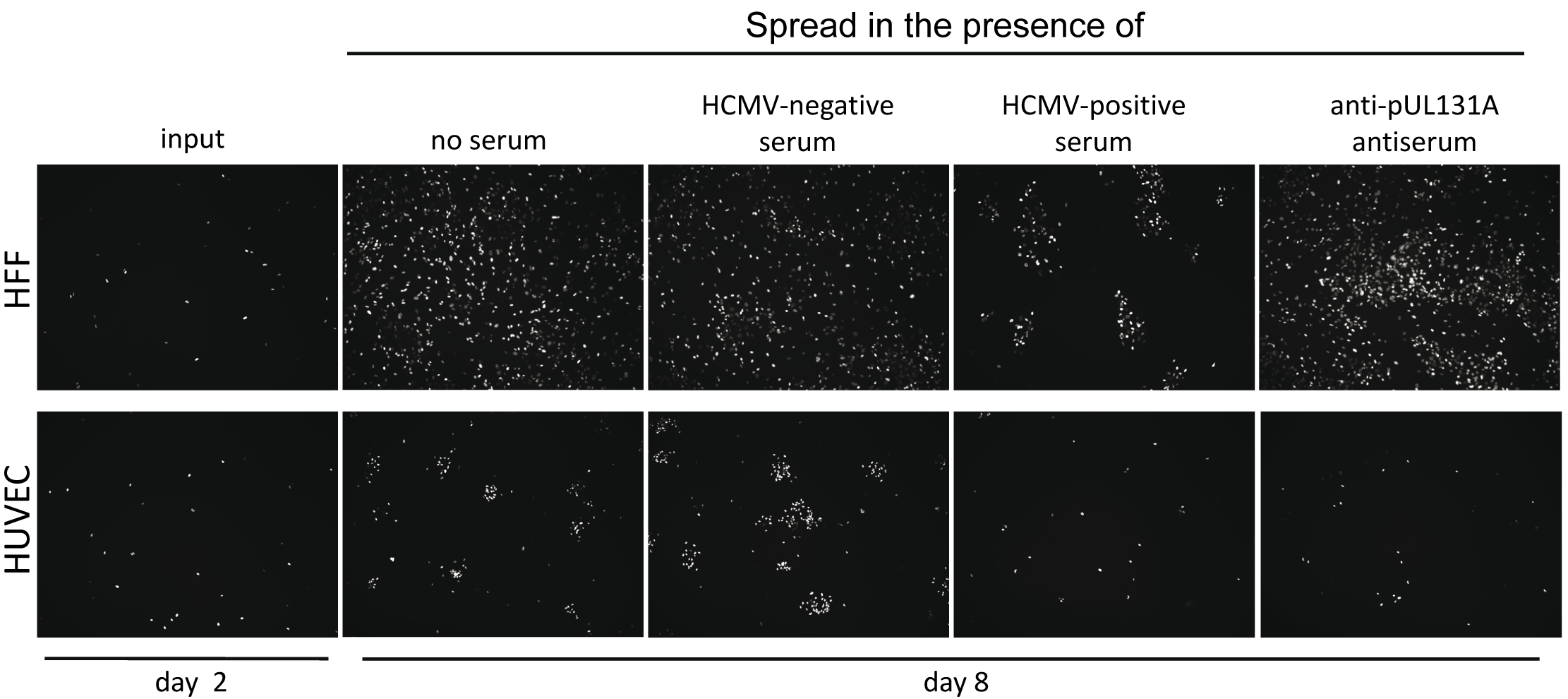

Supplement: Figure S1 — Neutralizing antibodies block focal spread of HCMV in EC cultures. HFF and HUVEC were infected with vBAC4-luc as described in Materials and Methods to obtain equal numbers of initially infected cells (m.o.i. on HFF: 0.1). After infection, cells were washed three times with medium and then fresh medium (no serum) or serum diluted in medium was added (1∶25 dilutions of the HCMV-negative and -positive sera and a 1∶10 dilution of the anti-pUL131A rabbit antiserum [17]). Cells were incubated in the presence of the antisera for 8 days. Initial infection (day 2) as well as virus spread (day 8) were monitored by staining for HCMV ie1 protein expression. The HCMV-positive antiserum showed a complete and the HCMV-negative serum no neutralization of HFF and EC infections when tested with free virus (data not shown). (0.56 MB TIF) [file ppat.1001256.s001.tif]

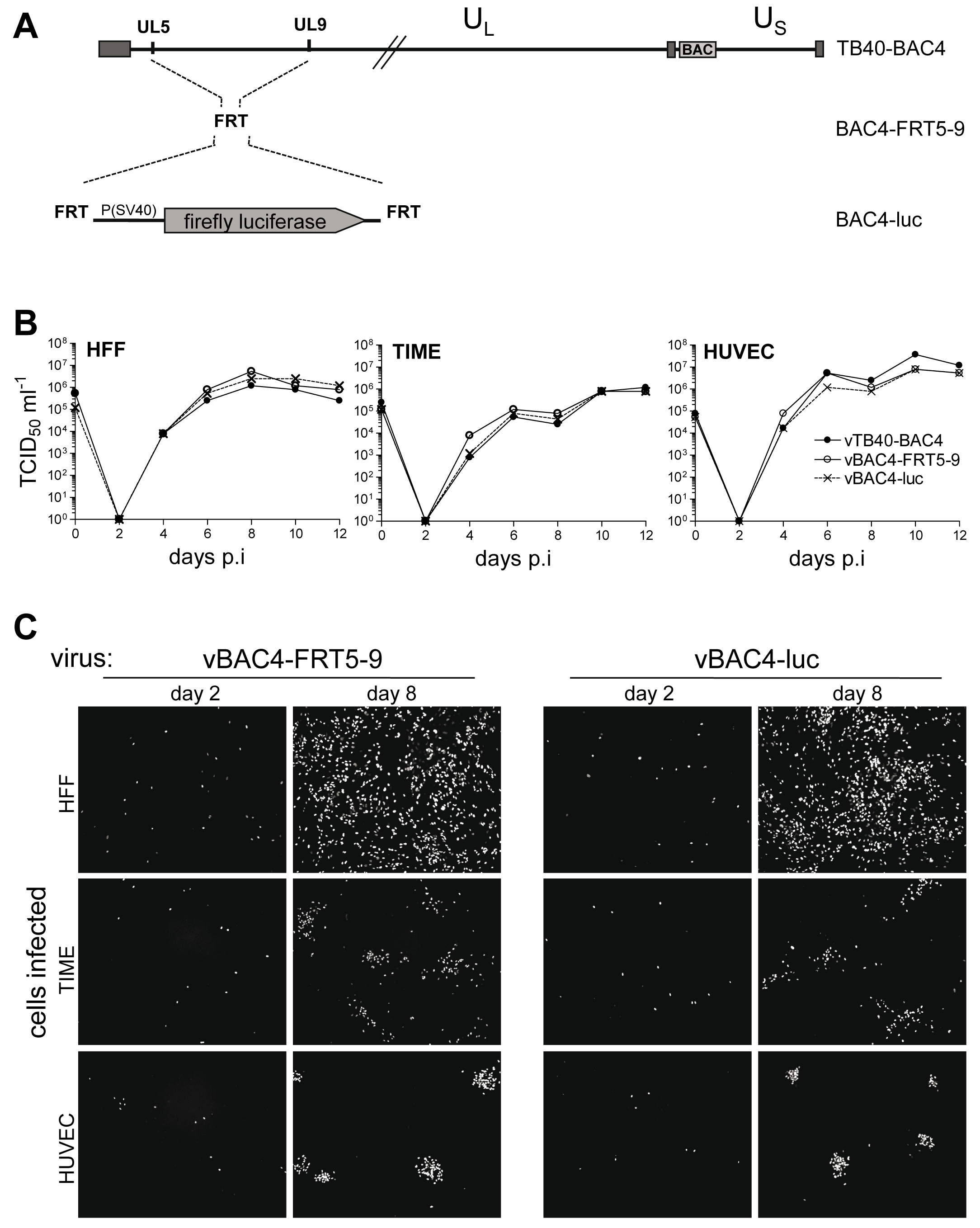

Supplement: Figure S2 — Growth properties of vBAC4-FRT5-9 and vBAC4-luc. (A) Schematic presentation of TB40-BAC4 derived mutants BAC4-FRT5-9 and BAC4-luc. The UL and US regions, the positions of internal and terminal repeats (dark grey), the open reading frames UL5 and UL9, the BAC cassette (light grey), the position of the FRT-site and the insertion of a luciferase expression cassette are indicated. (B) Growth curves of vTB40-BAC4, vBAC4-FRT5-9 and vBAC4-luc on HFF, TIME cells and HUVEC. Cells were infected as described in Materials and Methods to obtain equal numbers of initially infected cells (m.o.i. on HFF: 1). Cell culture supernatants were harvested at the indicated time points post infection and virus titers determined by a TCID50 assay performed on HFF. (C) Spread of vBAC4-FRT5-9 and vBAC4-luc in HFF and endothelial cell cultures. HFF were infected with vBAC4-FRT5-9 and vBAC4-luc at an m.o.i. of 0.1. For TIME cells and HUVEC the m.o.i. were adapted. The initial infections (day 2) as well as virus spread (day 8) were monitored by staining for HCMV ie1 protein expression. (0.63 MB TIF) [file ppat.1001256.s002.tif]
